# Interdisciplinary Approach for Management of Iatrogenic Internal Root Resorption: A Case Report

**DOI:** 10.7508/iej.2016.01.014

**Published:** 2015-12-24

**Authors:** Mohsen Ramazani, Saeed Asgary, Nafiseh Zarenejad, Javad Mehrani

**Affiliations:** a *Department of Endodontics, Sari Dental School, Mazandaran University of Medical Science, Sari, Iran;*; b *Iranian Center for Endodontic Research, Research Institute of Dental Sciences, Shahid Beheshti University of Medical Sciences, Tehran, Iran;*; c *Department of Restorative Dentistry, Sari Dental School, Mazandaran University of Medical Sciences, Sari, Iran;*; d *Department of Periodontics, Sari Dental School, Mazandaran University of Medical Sciences, Sari, Iran*

**Keywords:** Calcium-Enriched Mixture, Interdisciplinary Treatment, Internal Root Resorption

## Abstract

For management of a symptomatic maxillary lateral incisor with dull pain on chewing, suppurative sinus tract, defective metal-ceramic crown and iatrogenic internal root resorption, an interdisciplinary approach was taken. Two-visit nonsurgical treatment with calcium-enriched mixture (CEM) cement, replacement of metal-ceramic crown with all-ceramic crown and corrective periodontal plastic surgery were included in the treatment plan. Six-month and one-year follow-ups revealed complete resolution of signs and symptoms and radiographic healing. This case report highlights the importance of adequate cooling during crown preparation to preserve the pulp vitality and prevent internal resorptive lesions and also the profound sealing ability and biocompatibility of CEM cement.

## Introduction

Internal root resorption (IRR) is defined as the loss of dental hard tissues as a consequence of odontoclastic (dentinoclastic) activity, starting from the inner root canal wall. IRR is usually asymptomatic with slow progression and is detectible upon routine radiographic examination [1]. Many etiologic factors have been mentioned for IRR; incipient or recurrent caries, periodontal infections, traumas, excessive heat generated during crown preparation in vital teeth, insufficient remaining dentin thickness after preparation, marginal leakage of the crown, vital root resection, orthodontic treatment, cracks, idiopathic dystrophic changes within normal pulps and anachoresis [2-6]. 

In addition to the presence of granulation tissue in the pulp, damage to the odontoblastic layer and the predentin layer, lead to the pathologic entity by the adherence of clastic cells related to resorptive process [7]. Both the necrotic inflected pulp and the inflamed pulp can contribute to IRR [8]. Non-surgical root canal therapy to effectively remove the blood supply to the resorbing cells is conceptually the treatment of choice to inhibit the destructive pattern of the IRR [9]. When the process of root resorption has extended to the external root surface, the destruction of the regional periodontal tissues may occur and the situation is called perforating IRR [10]. 

There are different materials, in the market to treat the IRR. One of them is calcium-enriched mixture (CEM) cement, which is a water-based tooth-colored biomaterial [11, 12]. Several studies indicate that CEM cement has antibacterial and antifungal effects, that provides a physical and biological seal, non-toxicity and biocompatibility features and offers the advantage of inducing osteogenesis, dentinogenesis and cementogenesis [4, 11-14]. 

This case report describes an interdisciplinary management of a maxillary lateral incisor with perforating IRR including root canal therapy using CEM cement, replacement of the defective metal-ceramic crown by feldspathic porcelain crown, and periodontal plastic surgery for correction of the existing periodontal defect and elimination of the sinus tract related to IRR.

**Figure 1:**
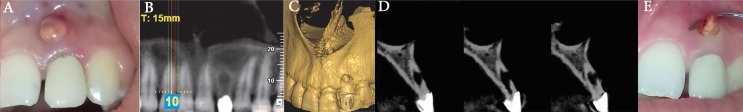
*A)* Suppurative fistula and defective PFM on tooth #22; *B)* Radiographic view of IRR; *C* and *D)* CBCT views of the tooth and adjacent bone destruction; *E)* Decrease of parulis size at the end of the second session due to irritants removal

**Figure 2:**
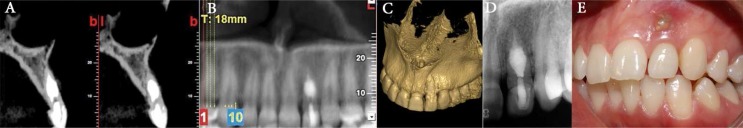
*A-D)* CBCT and radiographic views of CEM cement obturation, *E)* Cemented new all ceramic crown and the presence of sinus tract after one month

## Case Report

A 20-year-old female with her chief complaint being dull pain upon mastication on the left maxillary lateral incisor was referred to an endodontist. On clinical inspection the tooth was slightly tender to percussion and a suppurating sinus tract was obvious on the buccal gingiva of the tooth (Figure 1A). Periodontal probing depth was within the physiologic range (<3 mm) all around the tooth. Thermal and electrical tests failed to elicit a response. Radiographic examination revealed a well-defined, rather oval radiolucency between coronal and middle thirds of the root (Figure 1B). 

Cone-beam computed tomography (CBCT), was instructed and showed a perforating lesion leading to circular bone resorption with almost 7-8 mm diameter within the radicular bone (Figure 1C and 1D). The sinus tract was suppurative (Figure 1A). Based on the clinical and radiographic findings, the diagnosis was perforating IRR.

An interdisciplinary treatment strategy was designated. The following complications were decided to be covered; sealing of the perforative IRR, management of periodontal soft and hard tissues, providing coronal seal and esthetic reestablishment.

Patient signed the informed consent. Non-surgical root canal treatment was initiated after local administration of 2% lidocaine containing 1:80000 epinephrine (Darupakhsh, Tehran, Iran). The tooth was isolated with rubber dam. After preparation of the access cavity, the coronal part of the pulp was found to be necrotic, while bleeding was induced on middle part of the pulp. Working length was determined with radiography and canal instrumentation was conducted to master apical file (MAF) set to #40. Hand K-files (Dentsply Maillefer, Ballaigues, Switzerland) were used to thoroughly clean the pulp space and sever the blood supply. Irrigation was done with 1% sodium hypochlorite (Golrang, Pakshoo Co., Iran). At the end of the first session, calcium hydroxide (Aria Dent, Teheran, Iran) powder was mixed with saline to prepare the creamy intra-canal dressing. This would control the bleeding and facilitate the removal of remaining pulpal tissues during the second session. Access cavity temporization was done with light-cured glass ionomer (GI) cement (Fuji II LC, GC Corporation, Tokyo, Japan).

The second appointment was set 10 days later, when under rubber dam isolation, the remnants of pulpal tissue were removed by chemomechanical preparation. Elimination of tenderness on percussion, hemorrhage, discontinuation of suppuration through the sinus tract and decrease in the size of parolis confirmed the right time for obturation (Figure 1E). 

CEM Cement (BioniqueDent, Tehran, Iran) was used for canal obturation [15]. The whole canal space from apical constriction to 3 mm below the CEJ was filled with CEM cement which was carried into the canal with lentulo spiral (Dentsply, Maillefer, Ballaigues, Switzerland) and condensed with hand plugger (Figure 2A to D). A wet cotton pellet was put in the coronal part of the canal to provide the moisture needed for setting of coronal parts of the cement and coronal seal was achieved with GI cement.

After the completion of RCT, the patient was referred to a specialist of operative dentistry for crown preparation. After 1 month, the patient was asymptomatic, although the fistula was still present and the patient was unhappy with this situation (Figure 2E).

**Figure 3:**
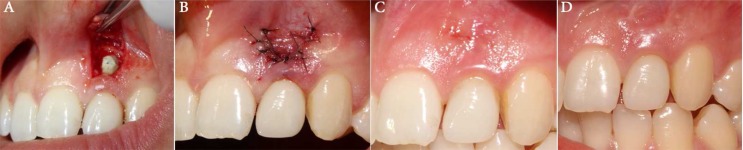
*A)* Periodontal flap to remove the granulation tissue and excess of the material; *B)* Immediate after surgery; *C)* 6-month follow up. Please note the progressing healing; *D)* One-year follow up showing complete healing

The patient had no systemic disease affecting tissue healing. The target area was anesthetized using lidocaine. A Luebke-Oschsenbein flap was prepared using a #15 surgical blade (Swann-Morton, Sheffield, England) to avoid the gingival margin and gain the benefits of a three-sided flap. The horizontal incision was carried out 3-4 mm away from the gingival margin and across the mid root extending bilaterally to the mesial and distal periodontium. Two diverging vertical releasing incisions were made at the ends of the horizontal incision extending to the alveolar mucosa to encompass the fistula. The flap was reflected using a periosteal elevator (H&H Corp., Ontario, CA, USA) beyond the area of bone destruction. The scar tissue was dissected from the flap with a blunt-tipped dissecting scissor (H&H Corp. Ontario, CA, USA). The granulation tissue, excess of CEM cement and the irregular borders of the perforation site were smoothened with a round bur (Thomas, Bourges Cedex, France) attached to an angulated surgical handpiece (Figure 3A). When the debridement was completed the flap was sutured with 7-0 nylon (Ethicon, NJ, USA) interrupted sutures (Figure 3B). Post-surgical recommendations were given to the patient and summoned her to return 2 weeks later for suture removal.

The patient remained symptom-free at one- and three-month follow-ups with good looking appearance of the gingiva, without periodontal pockets and sensitivity to percussion. After 6- (Figure 3C) and 12-month (Figure 3D) follow-ups, the patient was satisfied with the treatment from the esthetic point of view and also had no radiographic signs and clinical symptoms. 

## Discussion

This manuscript represented the successful outcome of a multidisciplinary approach for treatment of a maxillary incisor with perforative IRR. Respecting the principles for dentine preparation such as adequate cooling to preserve the pulp, it is postulated that increase of pulp temperature during crown preparation could probably be the reason for internal resorption in this case [16].

Although intraoral radiography is accurate enough, but superior level of accuracy obtained with CBCT increases the likelihood of correct management of internal resorption due to its ideal validity (ability to correctly detect the presence or absence of the lesion and its type) and reliability (ability to regenerate the similar results) [17-19]. In this case, CBCT was a trustable tool to accurately identify IRR and distinguish it from cervical root resorption, and also determine the lesion dimensions.

Successful treatment of IRR necessitates complete cleaning of pulpal space, severance of the blood supply, and filling the whole space with biocompatible materials especially when perforation has occurred [10, 20]. In the present case, non-surgical and orthograde obturation of the intra-pulpal space with CEM cement led to sufficient sealing of the borders between intra- and extra-pulpal spaces. This biomaterial sets faster than MTA and hence is not readily affected by the acidic inflammatory environment present in actively inflamed lesions like IRR [14]. CEM cement was also used because of its tooth colored formula, biocompatibility and providing apical and lateral seal of the perforation site [21].

Removal of irritation source should normally ameliorate the resultant parolis, though over extrusion of debris through the perforation site resulted in impairment of soft tissue healing. That is why periodontal intervention became necessary to correct the parolis. Corrective surgery approach allows practitioner to get direct access to the lesion and perform a mechanical cleaning of the target resorbed defect [22].

Complicated IRR is ought to be covered in interdisciplinary modality of treatment, because each part was managed by the most knowledgeable and experienced practitioner of the field.

## Conclusion

The interdisciplinary approach offered a systematic plan to manage perforating IRR and saved the tooth.
